# Serum Corticosterone and Insulin Resistance as Early Biomarkers in the hAPP23 Overexpressing Mouse Model of Alzheimer’s Disease

**DOI:** 10.3390/ijms22136656

**Published:** 2021-06-22

**Authors:** Jhana O. Hendrickx, Sofie De Moudt, Elke Calus, Wim Martinet, Pieter-Jan D. F. Guns, Lynn Roth, Peter P. De Deyn, Debby Van Dam, Guido R. Y. De Meyer

**Affiliations:** 1 Laboratory of Physiopharmacology, Faculty of Pharmaceutical, Biomedical and Veterinary Sciences, Campus Drie Eiken, Universiteitsplein 1, University of Antwerp, 2610 Wilrijk, Antwerp, Belgium; jhana.hendrickx@uantwerpen.be (J.O.H.); sofie.demooudt@uantwerpen.be (S.D.M.); wim.martinet@uantwerpen.be (W.M.); pieter-jan.guns@uantwerpen.be (P.-J.D.F.G.); lynn.roth@uantwerpen.be (L.R.); 2 Laboratory of Neurochemistry and Behaviour, Institute Born-Bunge, Department of Biomedical Sciences, Campus Drie Eiken, Universiteitsplein 1, University of Antwerp, 2610 Wilrijk, Antwerp, Belgium; elke.calus@uantwerpen.be (E.C.); peter.dedeyn@uantwerp.be (P.P.D.D.); debby.vandam@uantwerpen.be (D.V.D.); 3 Department of Neurology and Alzheimer Center, University of Groningen and University Medical Center Groningen, 9727 Groningen, The Netherlands

**Keywords:** hypercortisolism, insulin resistance, hypermetabolism, cognitive decline, Alzheimer’s disease

## Abstract

Increasing epidemiological evidence highlights the association between systemic insulin resistance and Alzheimer’s disease (AD). As insulin resistance can be caused by high-stress hormone levels and since hypercortisolism appears to be an important risk factor of AD, we aimed to investigate the systemic insulin functionality and circulating stress hormone levels in a mutant humanized amyloid precursor protein (APP) overexpressing (hAPP23+/−) AD mouse model. Memory and spatial learning of male hAPP23+/− and C57BL/6 (wild type, WT) mice were assessed by a Morris Water Maze (MWM) test at the age of 4 and 12 months. The systemic metabolism was examined by intraperitoneal glucose and insulin tolerance tests (GTT, ITT). Insulin and corticosterone levels were determined in serum. In the hippocampus, parietal and occipital cortex of hAPP23+/− brains, amyloid-beta (Aβ) deposits were present at 12 months of age. MWM demonstrated a cognitive decline in hAPP23+/− mice at 12 but not at 4 months, evidenced by increasing total path lengths and deteriorating probe trials compared to WT mice. hAPP23+/− animals presented increased serum corticosterone levels compared to WT mice at both 4 and 12 months. hAPP23+/− mice exhibited peripheral insulin resistance compared to WT mice at 4 months, which stabilized at 12 months of age. Serum insulin levels were similar between genotypes at 4 months of age but were significantly higher in hAPP23+/− mice at 12 months of age. Peripheral glucose homeostasis remained unchanged. These results indicate that peripheral insulin resistance combined with elevated circulating stress hormone levels could be potential biomarkers of the pre-symptomatic phase of AD.

## 1. Introduction

Alzheimer’s disease (AD) is the most common dementia syndrome, involving 50 million patients worldwide. Therefore, the need for earlier and more accurate detection of AD becomes critical. The earliest detectable changes in AD are neuropathological which are diagnosed by functional brain imaging, i.e., positron emission tomography (PET) [1] and magnetic resonance imaging (MRI) [2]. Additional diagnostic tests in clinical routine practice entail the use of the core AD cerebral spinal fluid (CSF) biomarkers, i.e., phosphorylated TAU, total TAU, and the Aβ_1–42_/Aβ_1–40_ ratio [3]. However, these diagnostic tools are both invasive and expensive and are usually only given to patients who are already showing clear symptoms of the disease. Despite all the efforts and advances made in recent decades to understand and address the pathogenesis of AD, no disease-modifying or preventive treatment options are currently available [4,5,6]. However, there is increasing epidemiological evidence of the association between metabolic and dementia syndromes [7,8,9,10,11]. More specifically, systemic insulin resistance is postulated to be an important factor in the pathophysiology of AD, a phenomenon also called type III diabetes mellitus (T3DM) [12,13,14,15,16,17]. Although insulin resistance was long considered a central feature of type II diabetes mellitus (T2DM), previous studies showed its contribution and presence in the AD brain in the absence of T2DM [17]. Additional studies in AD patients have demonstrated an association between insulin levels and cerebral amyloid deposition, and, importantly, hyperinsulinemia doubles the risk of developing AD [18,19]. Moreover, chronic glucocorticoid exposure is well known to result in whole-body insulin resistance [20,21,22]. Furthermore, patients with Cushing syndrome, an endocrine disorder of hypercortisolism, present with whole-body insulin resistance [23,24] and are more prone to develop a dementia-like phenotype [25,26,27,28].

While studies in experimental AD murine models have reported increased corticosterone levels [29], it is unclear whether changes in systemic insulin action and glucocorticoid exposure occur during the pre-symptomatic stage or around the time of observable clinical signs and subsequent diagnosis. Moreover, these studies largely do not cover the pre-symptomatic time-course of the AD pathophysiology. Given the seemingly increasing importance of circulating stress hormone levels and insulin resistance in the AD pathology, we aimed to investigate the systemic metabolic phenotype and circulating stress hormone levels in the well-known amyloidogenic hAPP23+/− overexpressing AD murine model longitudinally. In this way, we endeavored to study the possibility of stress hormone levels and insulin resistance as important biomarkers for people at risk of AD.

## 2. Results

### 2.1. Progressive Cognitive Decline with Increasing Age for hAPP23+/− Mice

**Figure 1:**
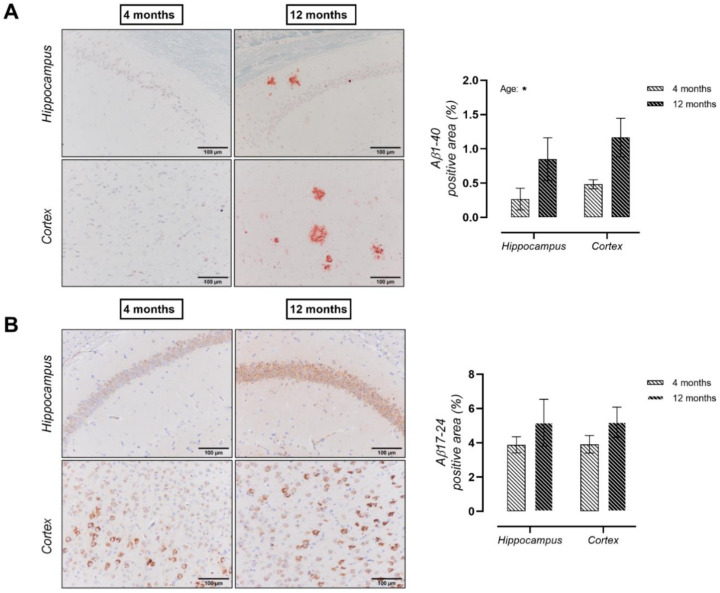
Assessment of cerebral amyloid peptide deposits. (**A**) Aβ_1–40_ deposits were significantly elevated in the hippocampus and parietal/occipital cortex of 12-month-old compared to 4-month-old hAPP23+/− brains (*n* = 6 for both groups at both ages; Factorial ANOVA for the factor ‘age’, * *p* < 0.05). (**B**) More Aβ_17–24_ peptide deposits were observed in the hippocampus and parietal/occipital cortex of 12-month-old compared to 4-month-old hAPP23+/− brains. (*n* = 6 for both groups at both ages; Factorial ANOVA for the factor ‘age’, *p* > 0.05).

**Figure 2:**
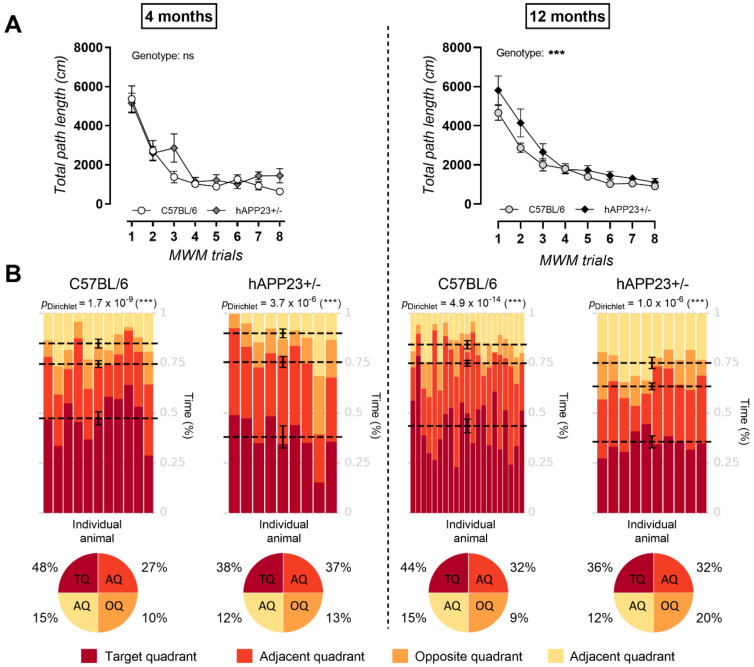
Morris-Water-Maze trial results. (**A**) At 4 months of age no genotypic differences were observed in total path lengths while at 12 months of age total path lengths differed significantly between genotypes (4 months, C57BL/6: *n* = 11; hAPP23+/−: *n* = 10 and 12 months, C57BL/6: *n* = 21; hAPP23+/−: *n* = 10; Factorial ANOVA for the factor ‘genotype’, *** *p* < 0.001). (**B**) At both ages hAPP23+/− animals displayed a poorer cognitive performance as compared to C57BL/6 mice during the MWM probe trial. Probe trial results deteriorated for hAPP23+/− animals from 4 to 12 months of age (4 months, C57BL/6: *n* = 11; hAPP23+/−: *n* = 10 and 12 months, C57BL/6: *n* = 21; hAPP23+/−: *n* = 10). Probe trial results were analyzed via Dirichlet distribution calculations as described earlier [27]. Each column represents the probe trial performance of an individual animal and each color represents a different quadrant of the MWM. Mean values for the fraction of time spent in each quadrant are represented by a dotted line with respective error bars for SEM. Average percentages of time spent by the animals in the MWM are indicated in the pie-chart beneath the heatmap of each tested group.

**Figure A1:**
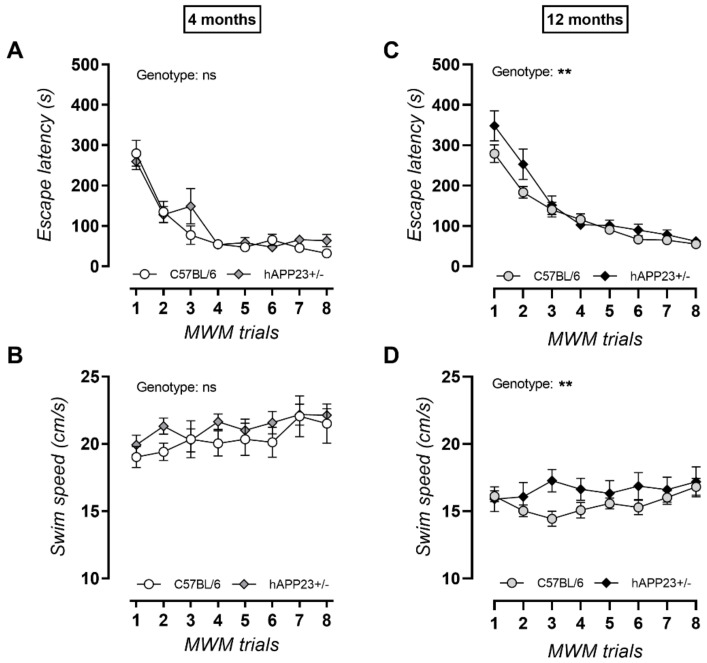
Escape latency and swim speed measurements during MWM acquisition trials. At 4 months of age, no genotypic difference was observed in terms of (**A**) escape latency and (**B**) swim speed. At 12 months of age hAPP23+/− showed prolonged (**C**) escape latencies despite significantly (**D**)| faster swim speeds compared to C57BL/6 littermates (4 months, C57BL/6: *n* = 11; hAPP23+/−: *n* = 10 and 12 months, C57BL/6: *n* = 21; hAPP23+/−: *n* = 10; Factorial ANOVA for the factor ‘genotype’, ** *p* < 0.01). These results underline the progressive neurodegenerative phenotype of hAPP23+/− animals.

Histological analysis for the presence of Aβ peptides in the hippocampus, parietal and occipital cortex of hAPP23+/− brains, revealed an increase in both Aβ1–40 (Figure 1A) and Aβ17–24 (Figure 1B) deposits at 12 months of age. Spatial learning and memory were evaluated via a MWM test. At 4 months of age, no clear performance differences could be observed during the acquisition trials between genotypes in terms of total path length (Figure 2A). However, at 12 months of age, a deteriorated performance was observed during acquisition trials for hAPP23+/− mice compared to C57BL/6 mice in terms of total path length (Figure 2A). Probe trial performance was further analyzed with Dirichlet distribution calculations [30] showing that hAPP23+/− mice exhibited reduced cognitive performance at both ages compared to C57BL/6 mice, which deteriorated with age (Figure 2B). Despite increased swim speeds, hAPP23+/− animals presented with longer escape latencies at 12 months of age compared to C57BL/6 control animals but not at 4 months of age (Figure A1).

### 2.2. Chronically Elevated Serum Corticosterone Levels in hAPP23+/− Mice

**Figure 3:**
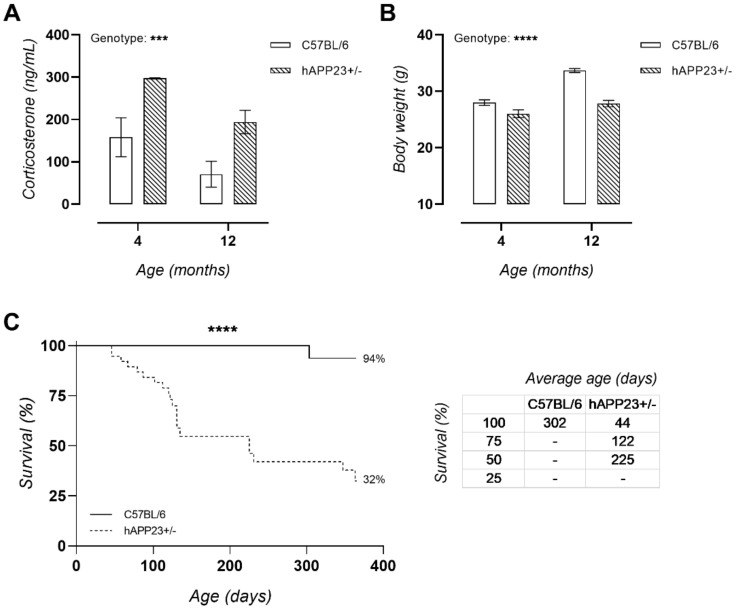
Serum corticosterone levels, survival, and body weight. (**A**) hAPP23+/− presented with increased serum corticosterone levels compared to C57/BL6J littermates (*n* = 5 for each group at 4 months of age and *n* = 9 for each group at 12 months of age; Factorial ANOVA for the factor ‘genotype’, *** *p* < 0.001). The SEM of the data of 4-months old hAPP23+/− animals are hardly visible. The respective data are 297 ±2 ng/mL. Furthermore, hAPP23+/− mice had a (**B**) lower body weight compared to C57BL/6 littermates (4 months, C57BL/6: *n* = 11; hAPP23+/−: *n* = 10 and 12 months, C57BL/6: *n* = 21; hAPP23+/−: *n* = 10; Factorial ANOVA for the factor ‘genotype’, **** *p* < 0.0001) and (**C**) survival rate (*n* = 38 for each group; Log-Rank Mantel-Cox test, **** *p* < 0.0001). The average age in days of the animals per genotype are listed in the table on the right per quartile percentage of survival.

Serum corticosterone levels were chronically elevated in hAPP23+/− mice (Figure 3A). In addition, hAPP23+/− mice presented with lower survival rates compared to C57BL/6 mice. At 12 months of age, only 32% of hAPP23+/− mice survived compared to 94% of C57BL/6 littermates (Figure 3B). Moreover, hAPP23+/− animals had significantly reduced body weights at both 4 and 12 months of age compared to C57BL/6 littermates (Figure 3C).

### 2.3. hAPP23+/− Display Decreased Urinary β-Hydroxybutyrate Levels Compared to C57BL/6 Mice

Equal urine volumes were observed between genotypes at both ages (4 months: 1.4 ± 0.1 mL vs. 1.1 ± 0.1 mL vs., 12 months: 2.3 ± 0.2 mL vs. 2.3 ± 0.1 mL; hAPP23+/− and C57BL/6 respectively). β-hydroxybutyrate levels were consistently decreased in hAPP23+/− mice as compared to control littermates at both ages (4 months: 0.13 ± 0.02 mM vs. 0.25 ± 0.04 mM; 12 months: 0.14 ± 0.03 mM vs. 0.18 ± 0.03 mM, *p* = 0.01; C57BL/6 and hAPP23+/− respectively) indicative of a metabolic shift from glucose metabolism to ketone body usage.

### 2.4. Unaltered Glucose Tolerance in hAPP23+/− Mice

**Figure 4:**
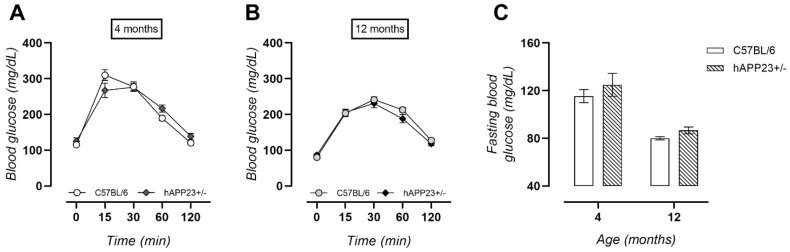
Glucose tolerance. (**A**) Similar tolerances were observed for hAPP23+/− and C57BL/6 mice (4 months, C57BL/6: *n* = 11; hAPP23+/−: *n* = 10 and 12 months, C57BL/6: *n* = 21; hAPP23+/−: *n* = 10; Factorial ANOVA for the factor ‘genotype’, *p* > 0.05). (**B**) At 12 months of age, glucose tolerances were equal (4 months, C57BL/6: *n* = 11; hAPP23+/−: *n* = 10 and 12 months, C57BL/6: *n* = 21; hAPP23+/−: *n* = 10; Factorial ANOVA for the factor ‘genotype’, *p* > 0.05). (**C**) No genotypic differences in fasting blood glucose levels could be observed (4 months, C57BL/6: *n* = 11; hAPP23+/−: *n* = 10 and 12 months, C57BL/6: *n* = 21; hAPP23+/−: *n* = 10; Factorial ANOVA for the factor ‘genotype’, *p* > 0.05) although values significantly decreased with age (4 months, C57BL/6: *n* = 11; hAPP23+/−: *n* = 10 and 12 months, C57BL/6: *n* = 21; hAPP23+/−: *n* = 10; Factorial ANOVA for the factor ‘age’, *p* < 0.001).

To investigate systemic glucose metabolism, a GTT was performed after a 16-h fasting period at 4 and 12 months of age. Similar glucose tolerances (Figure 4A,B) and corresponding fasting blood glucose levels (considered the blood glucose value measured at the start of the GTT) (Figure 4C) were obtained at both ages.

### 2.5. Pre-Symptomatic Insulin Resistance Evolves into Hyperinsulinaemia in hAPP23+/− Mice

**Figure 5:**
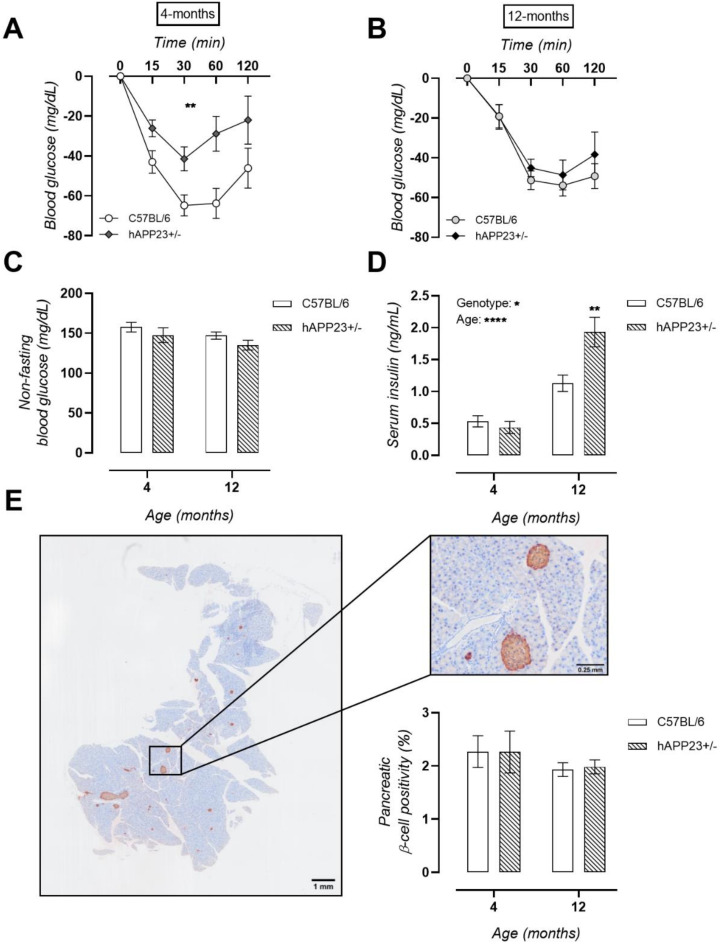
Insulin metabolism (**A**) Insulin resistance at the age of 4 months for hAPP23+/− mice compared to age-matched littermates (4 months, C57BL/6: *n* = 11; hAPP23+/−: *n* = 10 and 12 months, C57BL/6: *n* = 21; hAPP23+/−: *n* = 10; Factorial ANOVA for the factor ‘genotype’, ** *p* <0.01). (**B**) Similar insulin tolerances were observed for hAPP23+/− and C57BL/6 mice at 12 months of age (4 months, C57BL/6: *n* = 11; hAPP23+/−: *n* = 10 and 12 months, C57BL/6: *n* = 21; hAPP23+/−: *n* = 10; Factorial ANOVA for the factor ‘age’ and ‘genotype’, *p* > 0.05). (**C**) Non-fasting blood glucose levels remained unchanged between genotypes at both ages (4 months, C57BL/6: *n* = 11; hAPP23+/−: *n* = 10 and 12 months, C57BL/6: *n* = 21; hAPP23+/−: *n* = 10; Factorial ANOVA for the factor ‘age’ and ‘genotype’, *p* > 0.05). (**D**) Equal serum insulin levels at 4 months of age developed into hyperinsulinemia at 12 months of age for hAPP23+/− mice (4 months, C57BL/6: *n* = 11; hAPP23+/−: *n* = 10 and 12 months, C57BL/6: *n* = 10; hAPP23+/−: *n* = 9; Factorial ANOVA for the factor ‘age’ and ‘genotype’, * *p* < 0.05, **** *p* < 0.0001; with Sidak post-hoc test, ** *p* < 0.01). (**E**) Insulin staining of the pancreas resulted in equal β-cell positivity between genotypes (*n* = 6 for both groups at both ages, Factorial ANOVA for the factor ‘age’ and ‘genotype’, *p* > 0.05).

Peripheral insulin functionality was examined with an ITT at 4 and 12 months of age. At 4 months of age, significantly higher blood glucose levels were observed over the complete time-course of the ITT for hAPP23+/− mice, indicating peripheral insulin resistance (Figure 5A). This metabolic phenotype normalized at 12 months of age with equal systemic insulin tolerances between genotypes (Figure 5B). Overall, non-fasting blood glucose levels remained unchanged (Figure 5C). Equal serum insulin levels were measured at 4 months of age, which were, however, significantly increased at 12 months of age for hAPP23+/− mice compared to C57BL/6 mice, indicative of hyperinsulinemia in this murine model (Figure 5D). Pancreatic β-cell areas were determined by histological insulin staining. Although hAPP23+/− showed markedly different systemic insulin functionality, similar β-cell areas were observed (Figure 5E).

## 3. Discussion

Because the onset of the AD pathophysiology occurs years if not decades before the onset of noticeable clinical symptoms [31], the identification of early biomarkers is crucial. In the present study, we used the hAPP23+/− AD mouse model that shows progressive cerebral amyloidosis from young adulthood (4 months) to middle-age (12 months) [32] and significant cognitive decline at 12 months of age as demonstrated with the MWM test [33,34]. In addition to this pathological aging phenotype, we report a chronic state of stress in male hAPP23+/− mice, represented by chronically elevated circulating corticosterone levels, associated with lower body weights and survival rates. Elevated corticosterone levels have previously been measured in APP/PS1 transgenic mice [29] but were not followed up in time. Moreover, decreased body weights were previously observed in the same hAPP23+/− breeding line at our institute [35], as well as in other AD mouse models such as the Tg2576, APP/PS1, and TgCRND8 transgenic mice [36,37] and AD patients [38]. Importantly, stress is known to activate the hypothalamic-pituitary-adrenal (HPA) axis, leading to rapid synthesis and release of glucocorticoids (cortisol in humans and corticosterone in rodents) into the bloodstream. Hypercortisolism has been reported as a risk factor for AD [29,39,40,41,42] because it encompasses decreased brain structure volumes [26,43,44], impaired neuronal function, and drives neurons to cell death [45]. Accordingly, subclinical Cushing syndrome, characterized by elevated cortisol levels, has been reported as a potential cause of metabolic dementia and rapidly progressive Alzheimer-type dementia [25,27,28,39].

Although most findings report that chronic stress plays a role in the pathophysiology of AD, the question of why stress exacerbation occurs specifically in AD remained. Justice et al. [46] found the direct excitability of corticotrophin-releasing factor (CRF) neurons in cellulo when treated with soluble Aβ-species. Because cells can produce extracellular Aβ-species, which by themselves can acutely trigger and activate hypothalamic CRF neurons, it is conceivable that CRF neurons can be activated by these toxic peptides [46], ultimately leading to HPA dysregulation. Furthermore, HPA dysregulation is also known to affect hypothalamic control of eating behavior and calorie intake. Previously, increased food intake was measured in hAPP23+/− mice at our institute [35]. The fact that hAPP23+/− transgenic mice weigh less and were reported to eat more compared to their non-transgenic littermates argues for a hypermetabolic state in these animals. Moreover, in *APP* transgenic mice, severe hypoglycemic reactions, unexpected weight loss, rapid death, and extreme weakness are known to occur upon calorie restriction [47,48], further indicating the presence of a hypermetabolic state in these transgenic AD mouse models. Similar findings were obtained in a mouse model of tau deposition [49] and a triple-transgenic mouse model of AD [50], in which the animals ate more yet weighed less than non-transgenic littermates. In both studies, the authors define the observations as a hypermetabolic state.

An important feature of hypermetabolism is insulin resistance, which itself can also be caused by chronic exposure to glucocorticoids [20,21,22,51,52]. Accordingly, patients with Cushing syndrome present with whole-body insulin resistance [23,24]. The relationship between insulin resistance and AD, also called T3DM [15,16], is well recognized [12,13,14] and a subject of ongoing research. In this perspective, a recent epidemiological study has reinforced the detrimental role of chronic stress in T3DM and neurodegeneration [53]. Although there is speculation about the causal or consequential effect of insulin on the progression of AD, it still remains unclear when changes in insulin functionality occur in the AD timeline [54]. In the present study, insulin resistance was only present in a pre-symptomatic phase of the disease, reinforcing the notion that insulin dysregulation enhances the pathophysiology of AD. In relation to this finding, we could not detect T2DM features in this experiment, despite the well-reported association between T2DM and AD [55,56]. However, we did observe a chronic reduction in urinary β-hydroxybutyrate levels. We presume that the reduction of urinary ketone bodies may indicate decreased excretion by the body and increased uptake and utilization of ketone bodies in target tissues, such as the brain, and thus a metabolic switch. Along this line, recent research confirms the use of β-hydroxybutyrate as a brain fuel [57] and that its use has neuroprotective effects [58].

Altogether, our findings highlight the importance of circulating serum cortisol levels in humans (the equivalent of corticosterone in rodents) and insulin resistance as potential pre-symptomatic biomarkers in the AD pathology. In daily clinical practice, insulin resistance can be diagnosed via blood tests, i.e., fasting plasma glucose (FPG) and A1C test [59], or via an oral GTT. The euglycemic insulin clamp technique is the gold standard in a research setting, but an intravenous GTT (IVGTT) and/or an ITT/insulin suppression test are frequently used because they are easy to perform [59]. The HOMA-IR (Homeostatic Model Assessment for Insulin Resistance, derived from fasting insulin and glucose) approximates insulin resistance in humans. Measurement of serum cortisol levels belongs to standardized serum blood tests. In summary, these findings are promising, as both biomarkers are easy to measure, non-invasive, and relatively inexpensive.

## 4. Materials and Methods

### 4.1. Animal and Tissue Collection

Male hAPP23+/− overexpressing mice, carrying the human *APP* gene with the Swedish mutation (KM670/671NL) on a C57BL/6J background, were tested at 4 months (C57BL/6: *n* = 11; hAPP23+/−: *n* = 10) and 12 months (C57BL/6: *n* = 21; hAPP23+/−: *n* = 10) of age weighing approximately 28 ± 1 g (C57BL/6) and 26 ± 1 g (hAPP23+/−) at 4 months and 34 ± 1 g (C57BL/6) and 28 ± 1 g (hAPP23+/−) at 12 months of age. The expression of human *APP* is controlled by the murine neuronal *Thy1* promotor, resulting in an approximate tenfold overexpression of *APP* in heterozygous compared to control C57BL/6 mice [60]. hAPP23+/− mice together with their wildtype (WT) C57BL/6 littermates were bred and housed in the animal facility of the University of Antwerp. Genotypes were confirmed by polymerase chain reaction (PCR) (hAPP forward primer: CCGATGGGTAGTGAAGCAATGGTT; hAPP reverse primer: TGTGCCAGCCAACACAGAAAAC) on DNA of 4-week-old mice. All mice were socially housed in standard mouse cages with a maximum of eight animals per cage under conventional laboratory conditions with a constant room temperature (22 ± 2 °C), humidity (55 ± 5%), and artificial day/night cycle of 12 h/12 h (lights on at 8 a.m.). Food and water were provided *ad libitum*. All animal experiments were approved by the Animal Ethics Committee of the University of Antwerp (ECD n° 2017/53, approved on 26 July 2017) and conducted in accordance with the Guide for the Care and Use of Laboratory Animals, published by the National Institutes of Health (NIH Publication No. 85-23; Revised, 1996).

During the first week of the experiment, the learning and memory capability of animals was assessed via the MWM test. Next, urine was collected by individually housing the animals in metabolic cages (Tecniplast, Milan, Italy; floor area, 200 cm^2^) for 24 h. Next, metabolic testing of glucose and insulin tolerance was tested. At the end of the experiment, mice were humanely killed by perforating the diaphragm while under deep anesthesia (sodium pentobarbital (Sanofi, Machelen, Belgium), 250 mg/kg, ip [61]). Blood was collected from the retro-orbital plexus at sacrifice and subsequently centrifuged at 2000 rpm (4 °C for 10 min). Serum was stored at −20 °C until assayed. Pancreas and brain tissues were collected and immediately fixed in 4% formaldehyde for further histological analysis. Corticosterone and insulin levels were measured in serum using a highly sensitive corticosterone ELISA kit (EIA kit, Enzo Life Sciences, Farmingdale, NY, USA) and an insulin ELISA kit (80-INSMS-E01; ALPCO Diagnostics, Salem, NH, USA) according to the manufacturer’s instructions. The levels of the ketone body, β-hydroxybutyrate, were measured in urine samples using the colorimetric β-hydroxybutyrate (beta HB) Assay Kit (Abcam, ab83390; Cambridge, UK) according to the manufacturer’s protocol.

### 4.2. Spatial Learning and Memory

Memory and spatial learning were performed at the ages of 4 and 12 months with a MWM test. Compared to the classic protocol [34,62], we performed a ‘fast’ version consisting of two acquisition trial blocks daily with an interval of 4 h on four consecutive days. The trajectories of the mice were recorded using a computerized video tracking system (Ethovision, Noldus, Wageningen, The Netherlands) logging path length, escape latency, and swimming speed. Four days after the final acquisition trial block, a probe trial was performed. The platform was removed from the maze, and each mouse was allowed to swim freely for 100 s. Spatial accuracy was expressed as the percentage of time spent in each quadrant of the MWM, i.e., the specific location of the platform during the acquisition phase. Experimenters were blinded as to the genetic status of all animals.

### 4.3. Glucose and Insulin Tolerance Test (GTT, ITT)

Body weight and metabolic tests were performed one week before sacrifice. Glucose tolerance was determined after a 16-h fasting period by an intraperitoneal glucose injection (1 g/kg). Insulin tolerance was determined in a non-fasting state by an intraperitoneal injection of insulin (Novorapid, 1 U/kg; Novo Nordisk, Bagsværd, Denmark) (Heikkinen et al., 2007; Muniyappa et al., 2008). Fasting and non-fasting blood glucose levels were analyzed with a hand-held glucometer (OneTouch Verio^®^glucometer, range, 20–600 mg/dL; Lifescan, Milpitas, CA, USA) by taking a drop of venous blood from the tip of the mouse’s tail. Blood glucose levels were determined and plotted as a function of time up to 120 min after injection.

### 4.4. Histological Analysis

After euthanasia, pancreatic and brain tissues were fixed in 4% formaldehyde (BDH Prolabo, VWR Chemicals; West Chester, PA, USA) for 24 h, dehydrated overnight in 60% isopropanol (BDH Prolabo, VWR Chemicals; West Chester, PA, USA) and embedded in paraffin. Serial cross-sections (5 µm) of the respective tissues were prepared for histological analysis. All images were acquired using the Universal Grab 6.1 software with an Olympus BX40 microscope (Leica, Wetzlar, Germany) and were quantified using the Image J software (NIH, Bethesda, MA, USA). To study cerebral amyloidogenic progression, cerebral amyloid deposits were determined in the hippocampus and the parietal and occipital cortex by staining brain tissue with an anti-β-amyloid 17–24 (Biolegend, SIG-39200; San Diego, CA, USA; 1:200 dilution) and anti-β-amyloid 40 (Sigma, A8326; Burlington, NJ, USA; 1:1000 dilution) antibody, respectively. Positively stained percentage area fractions were calculated on three hippocampal and five cortical (parietal and occipital cortex) microscopic images of approximately 0.24 mm^2^. Pancreatic β-cell areas were evaluated by staining pancreatic sections for insulin (Abcam, ab181547; Cambridge, UK; 1:100,000 dilution). Fractions of positively stained β-cell areas were calculated relative to the total tissue area.

### 4.5. Statistical Analysis

Data are presented as mean ± SEM unless otherwise indicated. Overall, a factorial ANOVA test was performed for the factor’s ‘genotype’, ‘time’, and ‘interaction’ (genotype × time). A Shapiro–Wilk test and skewness were consulted to check the normality of data. The Levene’s test was used to check the equality of variances. Survival rates were analyzed with a Log-Rank Mantel–Cox test and statistical analysis of probe trial results were analyzed using Dirichlet distributions as described earlier [30]. Differences between genotypes were considered significant at *p* < 0.05. Applied statistical analyses are indicated in the figure legends and were performed using GraphPad Prism (version 8.0.0 for Windows, GraphPad Software, San Diego, CA, USA). Experimental sample sizes were determined as requested by the Ethical Committee of the University of Antwerp (file ECD 2017-53). To determine the sample size for the experiments, we used a power and sample size calculator (http://www.statisticalsolutions.net/pssZtest_calc.php (accessed on the 1 July 2017)) in combination with the expertise of the labs in relationship to the animal models used. Individual cohort sizes of 10 mice per time point gave power of 0.89 for an unpaired t-test analysis (assessed using PS: Power and Sample Size v 3.1.2–Vanderbilt University).

## 5. Conclusions

Our data show the pre-symptomatic presence of peripheral insulin resistance in hAPP23+/− mice, in the absence of T2DM, which eventually evolves into hyperinsulinemia in a symptomatic phase of the AD pathogenesis. In addition, we found an association between the stress hormone corticosterone and insulin resistance as a feature of hypermetabolism in these animals. These observations indicate the presence of elevated stress hormone levels, insulin resistance, and other observable hypermetabolic features as potential non-invasive biomarkers for the early detection of the AD pathophysiology. Possible limitations in this study are the relatively small sample size and the fact that only male mice were used.

**Figure 6:**
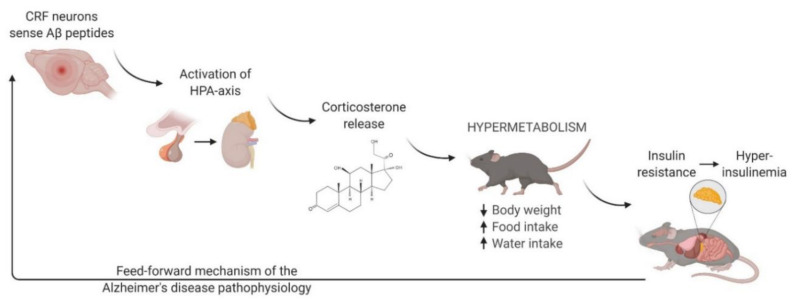
Proposed mechanism of amyloid-β induced corticosterone release, hypermetabolism, and insulin dysregulation. Following the secretion of toxic cerebral Aβ peptides, CRF neurons activate the HPA-axis, releasing corticosterone. As a result, a basal hypermetabolic state is induced, characterized by lean body weight and increased food and water intake. Over time, insulin resistance develops as a symptom of hypermetabolism, which in turn evolves into hyperinsulinemia upon chronic stress exposure. Eventually, a detrimental feed-forward mechanism of the AD pathophysiology develops. This figure was created with BioRender (www.biorender.com (accessed on 4 May 2021)).

In summary, we hypothesize that CRF neurons are activated upon the cellular secretion of toxic cerebral Aβ peptides, causing the HPA-axis to induce a generalized stress response and to release corticosterone (Figure 6). Chronic exposure to circulating corticosterone levels leads to hypermetabolism characterized by lean body weight, increased calorie intake, and insulin resistance. Prolonged insulin resistance eventually develops into hyperinsulinemia, which deteriorates glucose metabolism in the brain and forces the body to switch to ketone bodies. Altogether, a detrimental vicious cycle in AD pathophysiology ensues. In conclusion, our study identifies serum cortisol levels and insulin resistance as potential early biomarkers of AD.

## Data Availability

The data presented in this study are available on request from the corresponding author.
